# Relative overhydration is independently associated with left ventricular hypertrophy in dialysis naïve patients with stage 5 chronic kidney disease

**DOI:** 10.1038/s41598-020-73038-8

**Published:** 2020-10-02

**Authors:** Byoung-Geun Han, Jun Young Lee, Seung Ok Choi, Jae-Won Yang, Jae-Seok Kim

**Affiliations:** Department of Nephrology, Yonsei University Wonju College of Medicine, Kang-won, Wonju, Korea

**Keywords:** Biomarkers, Cardiology, Nephrology

## Abstract

Patients with chronic kidney disease (CKD) have a high prevalence of left ventricular hypertrophy (LVH), which increases as kidney function decreases. LVH pathophysiology is complex, making it difficult to generalise its evolution in CKD. Therefore, early detection and prevention of risk factors are critical. Assessment and management of volume status can minimise cardiovascular complications including LVH. We retrospectively investigated the associations between fluid overload and LVH in patients with stage 5 CKD not undergoing dialysis in prospective cohort of 205 patients (age: 59.34 ± 13.51 years; women: 43.4%). All patients, free of intrinsic heart disease, were assessed for relative overhydration/extracellular water (OH/ECW) by bioimpedance spectroscopy. Our results show that markers reflecting fluid balance were significantly higher in the LVH group and as OH/ECW increased, the left ventricular mass index (LVMI) trended higher. Furthermore, our results show that systolic blood pressure, serum phosphorus levels, and OH/ECW were independently associated with LVMI and that OH/ECW was independently associated with LVH. Structural and functional evaluation of the heart using echocardiography and volume status assessment using bioimpedance should be performed simultaneously in patients with early-stage CKD, even in those without evident cardiovascular disease.

## Introduction

Cardiovascular complications are the major cause of mortality and morbidity in patients with end-stage renal disease (ESRD). Structural and functional cardiac changes, such as left ventricular hypertrophy (LVH) and left ventricular (LV) diastolic dysfunction, can be seen in patients with even mild-to-moderate chronic kidney disease (CKD) regardless of the cause. Patients with CKD have a high prevalence of LVH, ranging from 29 to 74% in different studies, and its prevalence increases as kidney function decreases^1–7^.

However, the pathophysiology of LVH has not been completely elucidated in patients with gradually decreasing renal function due to the complexity of traditional and non-traditional cardiovascular risk factors. The clinical course and patterns of LVH are also determined by the stage of renal dysfunction. Previous studies regarding LVH have focused on patients with CKD who have a wide range of kidney function or patients with ESRD undergoing dialysis^8–13^.

The assessment and management of volume status are especially important as it can promote hemodynamic stability, minimise cardiovascular complications including LVH, and reduce the risk of cardiovascular events in patients with CKD^14^. LVH is a strong predictor of the risk of poor cardiovascular and renal outcomes^15–17^. Since LVH has been observed even in early-stage CKD and was associated with fluid overload, an early therapeutic approach to correct volume status has been suggested^18^. Bioimpedance is a non-invasive, quick, and relatively affordable method to quantitatively assess the volume status of a patient, although only a small number of studies have examined the relationship between volume status and LVH^11,12,19^.

An analysis regarding estimated glomerular filtration rate (eGFR) in a homogenous population is required. Furthermore, a sensitive therapeutic marker indicating precisely how much fluid overload is associated with LVH risk is required. Therefore, in this study, we particularly focused on the relationship between actual fluid overload and LVH in patients with stage 5 CKD not undergoing dialysis (CKD5-ND).

## Results

### Characteristics of the study patients

**Figure 1:**
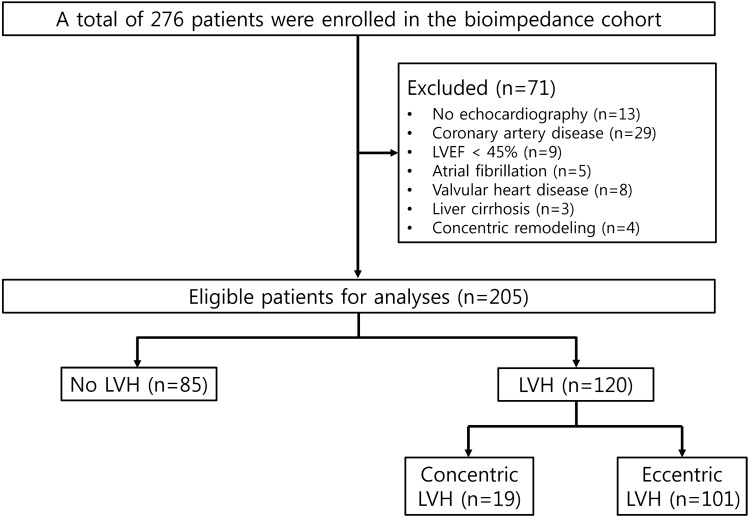
Flow diagram of patient selection in this study. *LVEF* left ventricular ejection fraction, *LVH* left ventricular hypertrophy.

Figure 1 depicts the flow diagram of the study. After the application of the exclusion criteria, a total of 205 patients (mean age, 59.34 ± 13.51 years; 43.4% females) were analysed. The clinical characteristics of each group according to the absence or presence of LVH are presented in Table 1. Among the total patients, 62.0% were aged below 65 years and 63.4% had diabetes. On echocardiography, a total of 120 patients (58.5%) had LVH (LVH group). LVH was more prevalent in women than in men (74.2 vs. 46.6%; *P* = 0.001). The systolic blood pressure (SBP) was significantly higher in the LVH group (*P* = 0.010). Among the echocardiographic parameters, the left atrial (LA) dimension (LAD), LA volume index (LAVI), E/e′ ratio, LV end-diastolic dimension (LVEDD), LV end-diastolic volume (LVEDV), left ventricular mass index (LVMI), and relative wall thickness (RWT) were substantially greater in the LVH group. Patients with LVH had lower levels of eGFR, albumin, calcium, and haemoglobin, but had considerably higher phosphorus levels. The levels of high-sensitivity C-reactive protein (hs-CRP) and intact parathyroid hormone (iPTH) did not differ between the two groups. Markers reflecting fluid balance such as overhydration (OH), OH/extracellular water (ECW), ECW/total body water (TBW), and N-terminal prohormone of B-type natriuretic peptide (NT-proBNP) were substantially higher in the LVH group.

**Table 1:** Comparison of demographics, serum chemistry, echocardiographic findings, and volume status between patients with and without left ventricular hypertrophy.

Variables	Total (n = 205)	No LVH (n = 85)	LVH (n = 120)	*P* value
Age, years	59.34 ± 13.51	58.52 ± 13.54	59.92 ± 13.52	0.466
**Age**				
< 65 years	127 (62.0%)	55 (43.3%)	72 (56.7%)	0.494
≥ 65 years	78 (38.0%)	30 (38.5%)	48 (61.5%)	
**Sex**				
Male	116 (56.6%)	62 (53.4%)	54 (46.6%)	0.001
Female	89 (43.4%)	23 (25.8%)	66 (74.2%)	
**Diabetes**				
Yes	130 (63.4%)	51 (39.2%)	79 (60.8%)	0.393
No	75 (36.6%)	34 (45.3%)	41 (54.7%)	
**Diuretics**				
Yes	130 (63.4%)	52 (61.2%)	78 (65.0%)	0.576
No	75 (36.6%)	33 (38.8%)	42 (35.0%)	
**RAASi**				
Yes	145 (70.7%)	58 (68.2%)	87 (72.5%)	0.509
No	60 (29.3%)	27 (31.8%)	33 (27.5%)	
**Phosphorous binders**				
Yes	31 (15.1%)	11 (12.9%)	20 (16.7%)	0.463
No	174 (84.9%)	74 (87.1%)	100 (83.3%)	
SBP, mmHg	142.19 ± 19.11	138.09 ± 20.08	145.08 ± 17.92	0.010
DBP, mmHg	80.42 ± 11.21	81.29 ± 12.22	79.80 ± 10.45	0.348
BMI, kg/m^2^	25.05 ± 4.12	24.89 ± 4.06	25.17 ± 4.18	0.635
LAD, cm	4.60 ± 0.46	4.45 ± 0.47	4.70 ± 0.43	< 0.001
LAVI, mL/m^2^	37.22 ± 10.05	32.11 ± 7.37	40.84 ± 10.14	< 0.001
E/e′ ratio	15.32 ± 5.29	13.36 ± 3.94	16.71 ± 5.69	< 0.001
LVEDD, cm	5.40 ± 0.46	5.27 ± 0.41	5.49 ± 0.47	< 0.001
LVEDV, mL	142.96 ± 28.35	134.61 ± 24.08	148.00 ± 29.73	< 0.001
LVMI, g/m^2^	114.72 ± 25.14	93.86 ± 13.29	129.50 ± 20.67	< 0.001
RWT	0.35 ± 0.06	0.33 ± 0.04	0.37 ± 0.06	< 0.001
LVEF, %	63.34 ± 5.56	63.00 ± 5.11	63.58 ± 5.86	0.467
NT-proBNP, pg/mL^a^	2,334 (657–8,014)	840 (351–5,803)	3,530 (1,329–10,102)	< 0.001
hs-CRP, mg/dL	1.31 ± 2.95	1.47 ± 2.91	1.19 ± 2.98	0.513
iPTH, pg/mL	301.18 ± 201.87	296.33 ± 227.71	304.65 ± 182.12	0.772
Haemoglobin, g/dL	9.04 ± 1.26	9.28 ± 1.24	8.87 ± 1.24	0.020
Total protein, g/dL	6.15 ± 0.77	6.36 ± 0.81	6.00 ± 0.70	0.001
Albumin, g/dL	3.49 ± 0.55	3.63 ± 0.53	3.39 ± 0.55	0.002
Total cholesterol, mg/dL	146.69 ± 40.12	140.91 ± 41.11	150.82 ± 39.05	0.082
HDL-C, mg/dL	38.34 ± 12.77	37.04 ± 12.74	39.27 ± 12.76	0.227
LDL-C, mg/dL	81.62 ± 35.30	76.22 ± 35.33	85.47 ± 34.93	0.070
Triglyceride, mg/dL	132.41 ± 72.70	139.16 ± 74.24	127.59 ± 71.50	0.263
Calcium, mg/dL	7.75 ± 1.06	7.95 ± 1.10	7.61 ± 1.00	0.023
Phosphorus, mg/dL	6.01 ± 1.54	5.61 ± 1.52	6.30 ± 1.49	0.001
eGFR, mL/min/1.73 m^2^	6.80 ± 2.46	7.58 ± 2.72	6.25 ± 2.10	< 0.001
OH, litre	2.89 ± 3.23	2.05 ± 2.85	3.49 ± 3.36	0.002
OH/ECW, %	15.02 ± 14.65	10.72 ± 14.11	18.11 ± 14.30	< 0.001
ECW/TBW	0.50 ± 0.05	0.49 ± 0.04	0.51 ± 0.05	< 0.001

*BMI* body mass index, *DBP* diastolic blood pressure, *ECW* extracellular water, *eGFR* estimated glomerular filtration rate, *HDL-C* high-density lipoprotein cholesterol, *hs-CRP* high-sensitivity C-reactive protein, *iPTH* intact parathyroid hormone, *LAD* left atrial dimension, *LAVI* left atrial volume index, *LDL-C* low-density lipoprotein cholesterol, *LVEDD* left ventricular end-diastolic dimension, *LVEF* left ventricular ejection fraction, *LVEDV* left ventricular end-diastolic volume, *LVH* left ventricular hypertrophy, *LVMI* left ventricular mass index, *NT-proBNP* N-terminal prohormone of B-type natriuretic peptide, *OH* overhydration, *RAASi* Renin–angiotensin–aldosterone system inhibitors, *RWT* relative wall thickness, *SBP* systolic blood pressure, *TBW* total body water.

^a^Mann-Whitney *U* test; Median (interquartile range).

**Figure 2:**
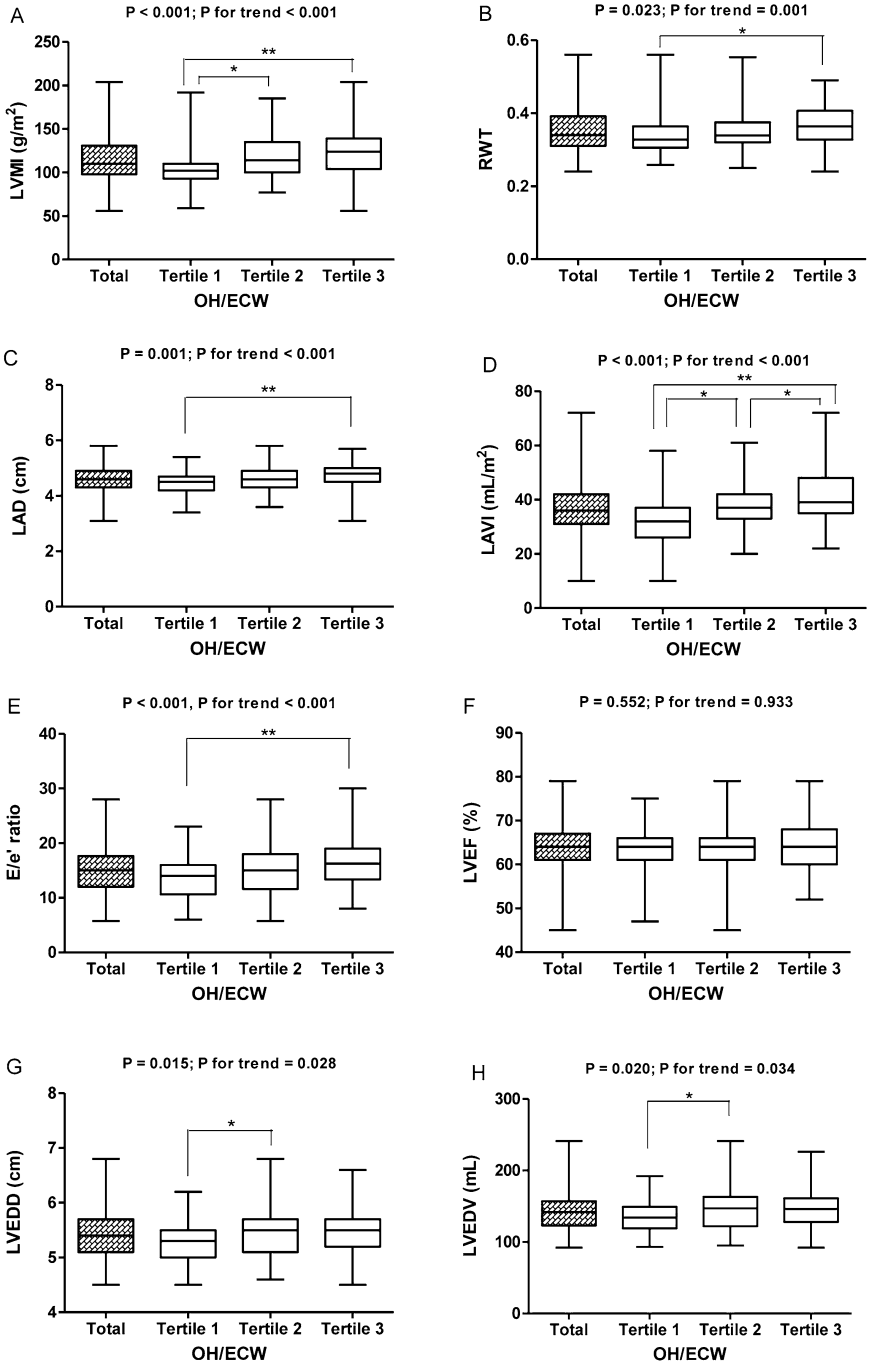
Left ventricular structural and functional alterations according to the distribution of OH/ECW tertiles. OH/ECW tertiles 1, 2, and 3 correspond to < 6.93, 6.93–19.66, and > 19.66%, respectively. *P < 0.05; **P < 0.001, as tested by one-way ANOVA with post hoc Bonferroni correction. *P* values for trend are also given. *ECW* extracellular water, *LAD* left atrial dimension, *LAVI* left atrial volume index, *LVEDD* left ventricular end-diastolic dimension, *LVEDV* left ventricular end-diastolic volume, *LVEF* left ventricular ejection fraction, *LVMI* left ventricular mass index, *OH* overhydration, *RWT* relative wall thickness.

The clinical characteristics of the patients according to the tertiles of OH/ECW are presented in Table 2. Compared with patients in the first tertile of OH/ECW, patients in the third tertile were more likely to have a history of diabetes, have a higher SBP, and have been treated with diuretics. There was a trend of higher levels of serum phosphorus, hs-CRP, and NT-proBNP across increasing tertiles of OH/ECW. In contrast, eGFR, haemoglobin, albumin, and calcium levels were lower in the third tertile group than in the first tertile group. Aggravation of the OH/ECW was substantially associated with echocardiographic findings, including LAD, LAVI, E/e′ ratio, LVEDD, LVEDV, LVMI, and RWT, while LV ejection fraction (LVEF) showed no difference between the three groups (Fig. 2).

**Table 2:** Comparison of demographics, serum chemistry, echocardiographic findings, and volume status according to OH/ECW tertiles.

Variables	OH/ECW (%)			*P* value	*P* for trend^#^
	Tertile 1	Tertile 2	Tertile 3		
Age, years	59.01 ± 14.21	60.91 ± 12.10	57.46 ± 14.22	0.339	0.383
**Age**					
< 65 years	41 (32.5%)	40 (31.7%)	45 (35.7%)	0.640	0.476
≥ 65 years	26 (34.7%)	27 (36.0%)	22 (29.3%)		
**Sex**					
Male	31 (27.2%)	44 (38.6%)	39 (34.2%)	0.073	0.164
Female	36 (41.4%)	23 (26.4%)	28 (32.2%)		
**Diabetes**					
Yes	31 (24.6%)	42 (33.3%)	53 (42.1%)	< 0.001	< 0.001
No	36 (48.0%)	25 (33.3%)	14 (18.7%)		
**Diuretics**					
Yes	38 (29.9%)	38 (29.9%)	51 (40.2%)	0.027	0.020
No	29 (39.2%)	29 (39.2%)	16 (21.6%)		
**RAASi**					
Yes	48 (34.0%)	46 (32.6%)	47 (33.3%)	0.931	0.851
No	19 (31.7%)	21 (35.0%)	20 (33.3%)		
**Phosphorous binders**					
Yes	7 (22.6%)	9 (29.0%)	15 (48.4%)	0.138	0.056
No	60 (35.3%)	58 (34.1%)	52 (30.6%)		
SBP, mmHg	134.18 ± 18.27	147.61 ± 18.22	145.01 ± 17.96	< 0.001	0.002
DBP, mmHg	78.57 ± 11.17	82.06 ± 11.77	81.06 ± 10.68	0.181	0.245
BMI, kg/m^2^	25.32 ± 3.84	24.70 ± 3.98	25.08 ± 4.62	0.691	0.489
LAD, cm	4.43 ± 0.42	4.61 ± 0.42	4.73 ± 0.49	0.001	< 0.001
LAVI, mL/m^2^	32.07 ± 8.41	37.57 ± 8.15	41.58 ± 10.79	< 0.001	< 0.001
E/e′ ratio	13.42 ± 3.61	15.03 ± 5.17	17.53 ± 6.12	< 0.001	< 0.001
LVEDD, cm	5.27 ± 0.40	5.48 ± 0.47	5.45 ± 0.48	0.015	0.028
LVEDV, mL	135.36 ± 23.49	147.78 ± 29.60	146.33 ± 30.00	0.020	0.034
LVMI, g/m^2^	104.28 ± 21.27	116.57 ± 23.01	123.55 ± 27.23	< 0.001	< 0.001
RWT	0.34 ± 0.05	0.35 ± 0.06	0.36 ± 0.06	0.023	0.001
LVEF, %	63.40 ± 5.25	62.78 ± 5.36	63.81 ± 5.87	0.552	0.933
NT-proBNP, pg/mL^a^	582 (318–1,665)	1,959 (748–5,077)	8,182 (4,350–20,855)	< 0.001	< 0.001
hs-CRP, mg/dL	0.69 ± 2.00	1.04 ± 2.68	2.13 ± 3.73	0.017	< 0.001
iPTH, pg/mL	325.17 ± 268.43	308.97 ± 173.62	276.44 ± 145.07	0.372	0.616
Haemoglobin, g/dL	9.55 ± 1.20	8.99 ± 1.22	8.64 ± 1.14	< 0.001	< 0.001
Total protein, g/dL	6.66 ± 0.63	6.18 ± 0.56	5.59 ± 0.71	< 0.001	< 0.001
Albumin, g/dL	3.85 ± 0.39	3.57 ± 0.44	3.03 ± 0.47	< 0.001	< 0.001
Total cholesterol, mg/dL	153.21 ± 40.01	135.33 ± 33.87	150.94 ± 44.46	0.019	0.553
HDL-C, mg/dL	38.65 ± 11.69	37.56 ± 12.35	38.83 ± 14.46	0.831	0.761
LDL-C, mg/dL	83.19 ± 36.05	75.32 ± 30.80	86.28 ± 38.82	0.190	0.781
Triglyceride, mg/dL	153.69 ± 97.62	115.70 ± 57.18	125.91 ± 50.22	0.007	0.191
Calcium, mg/dL	8.23 ± 1.17	7.69 ± 0.91	7.32 ± 0.88	< 0.001	< 0.001
Phosphorus, mg/dL	5.48 ± 1.29	6.19 ± 1.40	6.36 ± 1.75	0.002	0.001
eGFR, mL/min/1.73 m^2^	7.58 ± 2.36	6.65 ± 2.51	6.17 ± 2.23	0.003	0.001
OH, litre	0.04 ± 0.89	2.12 ± 0.74	6.51 ± 2.84	< 0.001	< 0.001
OH/ECW, %	0.04 ± 6.47	13.16 ± 3.74	31.86 ± 8.68	< 0.001	< 0.001
ECW/TBW	0.46 ± 0.03	0.49 ± 0.03	0.54 ± 0.03	< 0.001	< 0.001

OH/ECW tertiles 1, 2, and 3 correspond to < 6.93, 6.93–19.66, and > 19.66%, respectively.

*BMI* body mass index, *DBP* diastolic blood pressure, *ECW* extracellular water, *eGFR* estimated glomerular filtration rate, *HDL-C* high-density lipoprotein cholesterol, *hs-CRP* high-sensitivity C-reactive protein, *iPTH* intact parathyroid hormone, *LAD* left atrial dimension, *LAVI* left atrial volume index, *LDL-C* low-density lipoprotein cholesterol, *LVEDD* left ventricular end-diastolic dimension, *LVEF* left ventricular ejection fraction, *LVEDV* left ventricular end-diastolic volume, *LVMI* left ventricular mass index, *NT-proBNP* N-terminal prohormone of B-type natriuretic peptide, *OH* overhydration, *RAASi* Renin–angiotensin–aldosterone system inhibitors, *RWT* relative wall thickness, *SBP* systolic blood pressure, *TBW* total body water.

^#^*P* values obtained by the linear-by-linear association method or Jonckheere–Terpstra test.

^a^Kruskal–Wallis test; Median (interquartile range).

**Figure 3:**
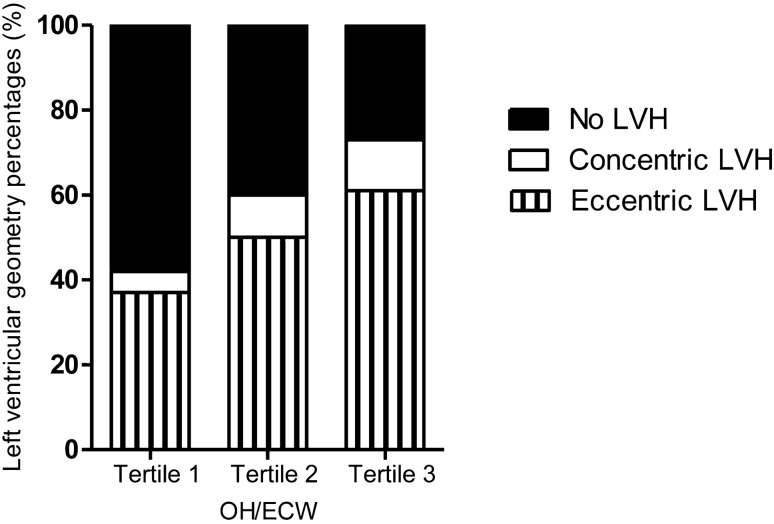
Percentage distribution of subjects according to the distribution of OH/ECW tertiles. OH/ECW tertiles 1, 2, and 3 correspond to < 6.93, 6.93–19.66, and > 19.66%, respectively. *P* for trend < 0.001, as tested by the linear-by-linear association method. *ECW* extracellular water, *LVH* left ventricular hypertrophy, *OH* overhydration.

As assessed by RWT and LVMI, the prevalences of eccentric LVH (eLVH) were 37.3%, 49.3%, and 61.2% and those of concentric LVH (cLVH) were 4.5%, 10.4%, and 11.9% in tertiles 1, 2, and 3 of OH/ECW, respectively, suggesting that eLVH progressively increased across increasing tertiles of OH/ECW (*P* for trend < 0.001) (Fig. 3).

### Multivariate analyses

In stepwise multiple linear regressions analyses, OH/ECW, SBP, and serum phosphorus were shown to be substantially associated with LVMI after adjustment for clinical confounding factors including age, prevalent diabetes, diuretics use, hs-CRP, albumin, and eGFR, while sex, body mass index (BMI), haemoglobin, serum calcium, and SBP were significantly associated with LVEDD (Table 3).

**Table 3:** Stepwise multiple linear regression of variables associated with left ventricular mass index and left ventricular end-diastolic dimension.

	LVMI^a^		LVEDD^a^	
	Β (95% CI)	*P* value	Β (95% CI)	*P* value
OH/ECW, %	0.321 (0.069, 0.573)	0.013		
Female (vs. male)			− 0.272 (− 0.393, − 0.151)	< 0.001
BMI, kg/m^2^			0.035 (0.021, 0.050)	< 0.001
SBP, mm Hg	0.222 (0.030, 0.415)	0.024	0.004 (0.001, 0.007)	0.012
Haemoglobin, g/dL			− 0.064 (− 0.114, − 0.015)	0.011
Calcium, mg/dL			− 0.078 (− 0.137, − 0.019)	0.010
Phosphorus, mg/dL	3.550 (1.249, 5.852)	0.003		

Only significant determinants were presented.

*B* β coefficient, *BMI* body mass index, *CI* confidence interval, *DM* diabetes mellitus, *ECW* extracellular water, *eGFR* estimated glomerular filtration rate, *hs-CRP* high-sensitivity C-reactive protein, *LVEDD* left ventricular end-diastolic dimension, *LVMI* left ventricular mass index, *OH* overhydration, *SBP* systolic blood pressure.

^a^Adjusted for: age, prevalent DM, diuretics use, hs-CRP, albumin, and eGFR.

When evaluating OH/ECW as a continuous variable, the association of OH/ECW with LVMI remained statistically significant. When evaluating OH/ECW as a categorical variable for further analysis, the substantial association between OH/ECW and LVMI disappeared in model 3, as did the other two variables (Table 4).

**Table 4:** Factors independently associated with left ventricular mass index.

Variable	Unadjusted	Model 1	Model 2	Model 3
	Β (95% CI)	Β (95% CI)	Β (95% CI)	Β (95% CI)
**OH/ECW analysed as a continuous variable**				
OH/ECW, %	0.360 (0.124, 0.596)	0.384 (0.145, 0.623)	0.475 (0.219, 0.731)	0.381 (0.029, 0.732)
SBP, mm Hg	0.223 (0.045, 0.400)	0.196 (0.014, 0.378)	0.196 (0.012, 0.380)	0.172 (− 0.035, 0.379)
Phosphorus, mg/dL	3.192 (1.007, 5.376)	3.373 (1.174, 5.572)	2.808 (0.568, 5.048)	2.270 (− 0.560, 5.100)
**OH/ECW analysed in tertiles**				
OH/ECW, %				
Tertile 1	Reference	Reference	Reference	Reference
Tertile 2	7.132 (− 1.321, 15.586)	6.954 (− 1.605, 15.514)	8.525 (− 0.080, 17.129)	7.110 (− 2.794, 17.013)
Tertile 3	14.146 (5.733, 22.559)	14.412 (5.942, 22.882)	16.336 (7.514, 25.158)	12.145 (− 0.180, 24.470)
SBP, mm Hg	0.223 (0.043, 0.403)	0.204 (0.021, 0.387)	0.209 (0.023, 0.395)	0.183 (− 0.025, 0.391)
Phosphorus, mg/dL	3.081 (0.889, 5.273)	3.263 (1.046, 5.480)	2.731 (0.462, 5.000)	2.218 (− 0.641, 5.077)

Model 1: Adjusted for age and sex.

Model 2: Adjusted for age, sex, BMI, prevalent DM, and use of diuretics, renin–angiotensin–aldosterone system inhibitors, and phosphorous binders.

Model 3: Adjusted for age, sex, BMI, prevalent DM, use of diuretics, renin–angiotensin–aldosterone system inhibitors, and phosphorous binders, haemoglobin, albumin, calcium, eGFR, and hs-CRP.

OH/ECW tertiles 1, 2, and 3 correspond to < 6.93, 6.93–19.66, and > 19.66%, respectively.

*B* β coefficient, *BMI* body mass index, *CI* confidence interval, *DM* diabetes mellitus, *ECW* extracellular water, *eGFR* estimated glomerular filtration rate, *hs-CRP* high-sensitivity C-reactive protein, *OH* overhydration, *SBP* systolic blood pressure.

When evaluating OH/ECW as a continuous variable in a multivariate logistic regression analysis, OH/ECW showed a substantial odds ratio in all three models. In addition, similar results were observed when OH/ECW was analysed in tertiles (Table 5). Serum phosphorus showed significant odds ratio in fully adjusted models, whereas the statistical significance of SBP disappeared in model 3. In all analytical models, the *P* value of the Hosmer–Lemeshow test exceeded 0.05.

**Table 5:** Multivariate analysis using logistic regression for predictive factors of left ventricular hypertrophy.

Variable	Model 1		Model 2		Model 3	
	OR (95% CI)	*P* value	OR (95% CI)	*P* value	OR (95% CI)	*P* value
**OH/ECW analysed as a continuous variable**						
OH/ECW, %	1.039 (1.013–1.065)	0.003	1.043 (1.015–1.071)	0.003	1.042 (1.008–1.076)	0.014
SBP, mm Hg	1.022 (1.004–1.041)	0.017	1.023 (1.004–1.042)	0.018	1.019 (0.998–1.040)	0.077
Phosphorus, mg/dL	1.413 (1.121–1.781)	0.003	1.423 (1.123–1.803)	0.003	1.482 (1.098–1.999)	0.010
**OH/ECW analysed in tertiles**						
OH/ECW, %						
Tertile 1	Reference		Reference		Reference	
Tertile 2	2.083 (0.900–4.823)	0.087	2.088 (0.894–4.875)	0.089	2.262 (0.870–5.883)	0.094
Tertile 3	3.839 (1.619–9.105)	0.002	4.122 (1.664–10.211)	0.002	3.907 (1.336–11.430)	0.013
SBP, mm Hg	1.023 (1.004–1.042)	0.015	1.024 (1.005–1.043)	0.015	1.020 (0.998–1.041)	0.072
Phosphorus, mg/dL	1.396 (1.106–1.763)	0.005	1.413 (1.113–1.794)	0.005	1.469 (1.087–1.985)	0.012

Model 1: Adjusted for age and sex.

Model 2: Adjusted for age, sex, BMI, prevalent DM, and use of diuretics, renin–angiotensin–aldosterone system inhibitors, and phosphorous binders.

Model 3: Adjusted for age, sex, BMI, prevalent DM, use of diuretics, renin–angiotensin–aldosterone system inhibitors, and phosphorous binders, haemoglobin, calcium, eGFR, and hs-CRP.

OH/ECW tertiles 1, 2, and 3 correspond to < 6.93, 6.93–19.66, and > 19.66%, respectively.

*CI* confidence interval, *BMI* body mass index, *DM* diabetes mellitus, *ECW* extracellular water, *eGFR* estimated glomerular filtration rate, *hs-CRP* high-sensitivity C-reactive protein, *OH* overhydration, *OR* odds ratio, *SBP* systolic blood pressure.

### Cut-off values of variables

Receiver operating characteristic (ROC) curves were drawn for OH, ECW/TBW, serum phosphorus, and OH/ECW to determine the cut-off values for predicting LVH. The optimal OH/ECW cut-off point for LVH was 10.050%, with a sensitivity of 69.2% and a specificity of 58.3% (Table 6).

**Table 6:** Cut-off values of volume markers and serum phosphorus for left ventricular hypertrophy.

	Cut-off point	AUC	SE	Sensitivity	Specificity	*P* value
OH, litre	1.550	0.636	0.040	69.2 (%)	56.0 (%)	0.001
OH/ECW, %	10.050	0.646	0.039	69.2 (%)	58.3 (%)	< 0.001
ECW/TBW	0.469	0.648	0.039	64.1 (%)	65.5 (%)	< 0.001
Phosphorus, mg/dL	5.400	0.657	0.039	72.6 (%)	56.0 (%)	< 0.001

*AUC* area under curve, *ECW* extracellular water, *OH* overhydration, *SE* standard error, *TBW* total body water.

## Discussion

Previous studies have illustrated that there are various clinical features of LV geometry in the CKD population^20^. An example is the wide heterogeneity in the prevalence of LVH, which may be due to the differences in the number of enrolled patients, the proportion of subjects, the presence of comorbidities, and whether or not the patient is undergoing dialysis treatment. It is also important to note that the inspection tools and diagnostic criteria used to diagnose LVH were not the same in each study.

As the pathophysiology of LV remodelling is very complex, it may be difficult to generalise the evolution of LVH with only one associated factor in patients with CKD. Although blood pressure still plays a major role in inducing LV remodelling in CKD, some other CKD-related factors are also known to be involved in this process. Some studies have proposed the independent risk factors for LVH in patients with CKD5-ND^7^, patients undergoing haemodialysis^21^, and patients undergoing peritoneal dialysis^12^. Chronic inflammation, anaemia, hypoalbuminaemia, hyperphosphataemia, arterial hypertension, and/or arterial stiffness, and high serum fibroblast growth factor 23 (FGF-23) levels were suggested as contributing factors for LVH in patients with CKD not undergoing dialysis^22–25^.

In the present study, the levels of hs-CRP, an inflammatory marker, tended to increase with increasing volume (*P* for trend < 0.001); however, there was no significant difference between the patients with or without LVH (*P* = 0.513) and there was no association with LVMI. In stepwise multiple linear regressions, haemoglobin and serum calcium levels were associated with LVEDD, but not with LVMI. Sex differences and the contribution of BMI to LVH development were not significant in our study (Table 2, Supplementary Table S1). The prevalence of LVH in the general population with diabetes is known to be high^26^, but there was no difference in the prevalence of LVH between patients with and without diabetes in our study (Table 1). This is probably due to a combination of factors other than diabetes, such as chronic inflammation, hypertension, anaemia, and fluid overload.

Increased serum phosphorus levels have been associated with an increased risk of cardiovascular mortality in patients with CKD stages 3–4^27^. Serum phosphorus is independently associated with LV mass in patients with CKD. The reasons for this association are unclear. However, previous studies had suggested possible causes such as vascular calcification, arterial stiffness, and increased ventricular workload in higher serum phosphorus^24,28^. In a multivariate logistic regression analysis, serum phosphorus levels were found to be substantially associated with LVH. This suggests that lowering phosphorus levels can be considered as one of the therapeutic goals toward improving cardiovascular outcomes associated with LVH from an early stage of CKD.

Arterial hypertension is independently associated with LVH in patients with CKD^1^. Although hypertension is not a reliable biomarker of volume overload in patients with ESRD, the effect of volume overload on blood pressure is crucial. LVH represents an adaptive response to volume and/or pressure overload^2^. It is well known that cLVH results from increased afterload such as hypertension, whereas eLVH is a consequence of increased preload such as fluid overload and/or renal anaemia^29^. We do not know exactly whether fluid overload directly or indirectly influences left ventricular remodeling in this study. Considering the high eLVH patient ratio in our study, it suggests that there is a limit to explaining that the development of LVH in patients with advanced CKD is simply caused by hypertension.

Fluid overload has been associated with cardiovascular morbidity and all-cause mortality in patients with CKD not undergoing dialysis^17,30,31^. The poor prognosis of fluid overload is mainly explained by the link with cardiovascular effects such as LVH, LV systolic and diastolic dysfunction, pulmonary hypertension, and increased aortic stiffness^32^. The relationship between fluid imbalance and LV remodelling has been primarily elucidated through studies involving patients on dialysis^11,12,19^. The association of fluid overload and cardiac dysfunction has been rarely reported in patients with advanced CKD^33,34^. Moreover, few studies have directly and objectively measured volume status and selectively compared volume status with LVH^18,35^. Volume status assessed by bioimpedance spectroscopy (BIS) was not independently associated with LV mass measured by magnetic resonance imaging in patients with mild-to-moderate CKD^5^. LVH was not associated with relative fluid overload in patients undergoing haemodialysis^36^. However, relative overhydration was independently associated with LVH in multivariate analyses in our study. The differences from the results of previously mentioned studies may be due to differences in the enrolment criteria, patient cohort composition, and diagnostic criteria for the analysis. NT-proBNP, a marker for fluid overload in our study, showed significantly different levels between patients with and without LVH (*P* < 0.001). NT-proBNP is known to be associated with LVH^37^. However, it is unclear whether NT-proBNP itself reflects volume status or whether it is a by-product of structural damage to the myocardium due to fluid overload.

This study includes several limitations. We did not perform stress echocardiography to exclude asymptomatic patients with underlying cardiac impairment such as ischaemic heart disease. We did not measure LV mass by cardiac magnetic resonance imaging. Although cardiac magnetic resonance imaging is the best way to define LV mass and patterns of LVH^29^, it is not used in routine practice in our hospital because it is more expensive than echocardiography and includes a risk for nephrogenic systemic fibrosis. Since our assessment was only performed at one point, we could not determine fluctuations in LV geometry^38^. Serial changes in cardiac structures related to the removal of overload fluid by renal replacement therapy could not be assessed over time. Therefore, the relationship between fluid overload and LVH does not allow us to evaluate causality in this observational study. In addition, although the degree of urine volume and proteinuria were factors that could affect our research, we could not use them for analysis because they were not perfectly measured in all patients. However, this study has some strengths. First, all patients were free of intrinsic heart disease and represented a relatively homogenous population in terms of eGFR. Second, prior to performing dialysis treatments that may affect fluid balance, volume status was objectively measured at the time of echocardiography.

Our study demonstrates the specific levels of overhydration and serum phosphorus that affect the development of LVH in patients with CKD5-ND. Regardless of CKD stage, we can effectively prevent LV remodelling and improve outcomes through the tailored application of treatment strategies aimed at the risk factors identified in this study. Therefore, our results suggest that structural and functional evaluation of the heart using echocardiography and volume status assessment using bioimpedance should be performed simultaneously in patients with early-stage CKD rather than only in patients with stage 5 CKD, regardless of evident cardiovascular disease. Further research is needed to validate the consistency of this association across other stages of CKD.

## Materials and methods

### Patients and data collection

From September 2014 to December 2019, we have registered consecutive patients with CKD5 to a bioimpedance cohort. Therefore, the current study was a retrospective observational analysis of a prospective cohort database. All patients were hospitalised to plan their first dialysis treatment. Patients registered to the cohort underwent BIS, echocardiography, and laboratory evaluation at the time of enrolment, prior to dialysis.

We excluded 51 patients with structural and functional cardiac abnormalities to reduce the effects of underlying heart disease that could cause LVH. We also excluded 3 patients with liver cirrhosis to minimise the impact of fluid imbalance on the study results. Patients who had a history of angina or myocardial infarction and patients who had findings of infarction on electrocardiography or had regional wall motion abnormalities on echocardiographic examination were considered as patients with coronary artery disease. Patients with concentric remodelling were excluded from the analysis because the total number of these patients was too small for analysis (n = 4) (Fig. 1).

This study was conducted in accordance with the Declaration of Helsinki. This study was initiated after receiving approval (no. CR316024) from the Institutional Review Board of Yonsei University Wonju Severance Christian Hospital. All patients provided written inform consent prior to participation in the study.

### Conventional echocardiographic study

Echocardiography was performed in the harmonic imaging mode using a 3-MHz transducer and commercial ultrasound system (GE Vivid E9; GE Healthcare, Chicago, IL USA). The LV mass was calculated following the American Society of Echocardiography recommendations using the following equation:

$$ {\text{LV}}\,{\text{mass}}\, = \,0.8\, \times \,\left\{ {1.04\, \times \,\left( {\left[ {{\text{PWTd}}\, + \,{\text{SWTd}}\, + \,{\text{LVDd}}} \right]^{3} \, \times \,\left[ {{\text{LVEDD}}} \right]^{3} } \right)} \right\}\, + \,0.6\,{\text{g}} $$

where PWTd and SWTd are the posterior and septal wall thickness at end-diastole, respectively, and the LVEDD is the M-mode LV dimension with the short axis view at end-diastole. To correct for body surface area, the LVMI was calculated by dividing LV mass by body surface area (BSA), using the formula as follows: BSA = (0.007184 × weight^0.425^ × height^0.725^) m^2^. The LA volume can be computed by using the area-length approximation: LA volume = [8/(3 π)][(A1 × A2)/L], where A1 and A2 are the corresponding LA areas measured in the apical two- and four-chamber views. The LA length L is defined as the shortest of the two long axes measured in each view. The LA volume index (LAVI) was calculated by dividing LA volume by BSA. LVEDV and LVEF were measured using the biplane modified Simpson’s rule, according to the previously mentioned recommendations. Transmitral early diastolic (E wave) velocities and the peak early (e′) diastolic mitral annular velocities at the septal mitral annulus were measured. We calculated the E/e′ ratio.

LVH was defined as LVMI > 95 g/m^2^ in women and > 115 g/m^2^ in men. RWT was calculated by the formula: RWT = (2 × PWT)/LVEDD. Patients were divided into 4 categories according to the RWT and the presence of LVH as follows: normal geometry (no LVH, RWT ≤ 0.42), concentric remodelling (no LVH, RWT > 0.42), eLVH (LVH, RWT ≤ 0.42), and cLVH (LVH, RWT > 0.42)^39^. Echocardiography was performed by trained cardiologists who were completely blinded to patient information.

### Assessment of the volume status

BIS was performed using the BCM (Body Composition Monitoring, Fresenius Medical Care AG & Co., Bad Homburg vor der Höhe, Germany) at enrolment prior to any renal replacement therapy. The validity of BIS in the general and dialysis populations has been demonstrated in comparison to gold standard methods. ECW, intracellular water, and TBW were automatically provided by BCM using equations of Moissl et al.^40^. OH level, OH/ECW, ECW/TBW, and NT-proBNP level are generally used as markers of fluid balance. Extracellular fluid overload, represented as OH, can be calculated from the difference between the actually measured ECW and the normally expected ECW^41^. As measurement of relative overhydration, OH/ECW was primarily used to determine volume status for our analysis.

### Laboratory evaluations

All laboratory assessments were also performed at enrolment. Complete blood cell count was collected using a Sysmex XE-2100 (Sysmex, Kobe, Japan). Clinical chemistry test items including creatinine, albumin, calcium, phosphorus, hs-CRP, total cholesterol, high-density lipoprotein cholesterol, low-density lipoprotein cholesterol, and triglyceride were measured using a Cobas 8000 Modular Analyzer (Roche Diagnostics GmbH, Mannheim, Germany). The eGFR from serum creatinine was calculated using the Modification of Diet in Renal Disease formula. The NT-proBNP level was measured using the Modular Analytics E170 System (Roche Diagnostics GmbH, Mannheim, Germany). The analytical measuring range of NT-proBNP was from 5 to 35,000 pg/mL. The hs-CRP level less than 0.3 g/dL (3 g/L) is normal.

### Statistical analysis

Categorical variables were reported as frequencies and percentages, and continuous variables were reported as means with standard deviations or medians with interquartile ranges, as appropriate. All patients were classified into two groups according to the absence or presence of LVH and were divided into three groups according to tertiles of OH/ECW for analysis. Differences in clinical variables between the two groups were tested with two-sample *t*-tests and Mann–Whitney *U* tests for continuous variables. The nominal variables were compared using a chi-squared test and Fisher’s exact test as appropriate. Patient characteristics were also compared across tertiles of OH/ECW using a chi-squared test, analysis of variance (ANOVA) with post hoc Bonferroni correction, and Kruskal–Wallis test. A linear-by-linear association method and a Jonckheere–Terpstra trend test were used for analysing trends in the OH/ECW tertiles. LVEDD, LVEDV, RWT, and LVMI can affect the LV geometry pattern. Therefore, we first performed stepwise linear regression analyses to identify the potential determinants of LV structural indices (LVMI, LVEDD) adjusted for age, prevalent diabetes, diuretics use, hs-CRP, albumin, and eGFR. Parameters significantly associated with LVMI or LVEDD (*P* < 0.05) were used preferentially in the multivariate regression analyses. Uses of anti-hypertensive drugs and phosphorous binders were also included in the multivariate models because of their established relationship with LVH despite the lack of statistical significance in our results. The covariates that had statistical significance in the univariate analysis and needed to be clinically considered were simultaneously entered into the multivariate models. We performed a multiple linear regression analysis to explore the association of LVMI with the identified determinants. Multivariate logistic regression models were used to examine the associations of OH/ECW, SBP, and serum phosphorus with LVH. The goodness of fit for model reliability was assessed using the Hosmer–Lemeshow test, whereby we considered a value of *P* < 0.05 to indicate that the model had a poor fit. OH/ECW was considered as both a continuous and categorical variable in the multivariate linear regression and multivariate logistic regression analyses. Finally, an ROC curve was created to establish cut-off values of OH, ECW/TBW, serum phosphorus, and OH/ECW that discriminate between patients with LVH and those without LVH. All analyses were performed with IBM SPSS Statistics software (version 23.0; IBM Corporation, Armonk, NY, USA). Graphs were generated with Prism software (version 5.02; GraphPad Software, San Diego, CA, USA). Statistically significant differences were defined as those having *P* values < 0.05.

## Supplementary information

Supplementary Table S1.
