# Supplementary material for: Relative overhydration is independently associated with left ventricular hypertrophy in dialysis naïve patients with stage 5 chronic kidney disease
Source: Sci Rep. 2020 Oct 2;10:15924. doi: 10.1038/s41598-020-73038-8 (PMC7532187; doi:10.1038/s41598-020-73038-8)
Supplement: Supplementary file 1 — Supplementary Table S1. [file 41598_2020_73038_MOESM1_ESM.pdf]

Relative overhydration is independently associated with left ventricular hypertrophy in dialysis naïve patients with stage 5 chronic kidney disease

Byoung-Geun Han, Jun Young Lee, Seung Ok Choi, Jae-Won Yang, and Jae-Seok Kim

Supplementary Table S1. Comparison of variables between males and females.

Variables	Male (n=116)	Female (n=89)	*P*-value
Age, years	59.24±13.99	59.46±12.93	0.909
SBP, mmHg	143.92±18.93	139.92±19.22	0.138
DBP, mmHg	80.78±11.05	79.96±11.47	0.605
BMI, kg/m^2	24.28±4.03	25.35±4.25	0.365
LAD, cm	4.63±0.52	4.56±0.39	0.320
LAVI, mL/m^2	37.24±10.09	37.20±10.05	0.978
E/e′ ratio	14.40±4.85	16.51±5.62	0.005
LVEDD, cm	5.52±0.44	5.23±0.44	<0.001
LVEDV, ml	150.61±27.85	131.81±29.31	<0.001
LVMI, g/m^2	115.80±24.84	113.31±25.59	0.484
RWT	0.35±0.05	0.36±0.06	0.374
LVEF, %	63.38±5.06	63.28±6.17	0.900
NT-proBNP, pg/mL*	2,030 (572 ~ 7,774)	2,687 (840 ~ 9,563)	0.302
hs-CRP, mg/dL	1.48±3.12	1.08±2.72	0.370
iPTH, pg/mL	260.10±123.54	355.33±264.01	0.002
Hemoglobin, g/dL	9.10±1.20	8.96±1.32	0.415
Total protein, g/dL	6.12±0.76	6.18±0.78	0.554
Albumin, g/dL	3.48±0.54	3.50±0.57	0.736
Total cholesterol, mg/dL	140.50±41.16	154.70±37.46	0.012
HDL-C, mg/dL	36.20±12.29	41.21±12.91	0.006

LDL-C, mg/dL	79.12±36.41	84.98±33.68	0.251
Triglyceride, mg/dL	129.82±68.69	135.76±77.84	0.564
Calcium, mg/dL	7.61±0.93	7.94±1.18	0.028
Phosphate, mg/dL	5.98±1.61	6.07±1.45	0.680
eGFR, mL/min/1.73 m^2	7.41±2.64	6.01±1.94	<0.001
OH, liter	3.31±3.20	2.34±3.20	0.034
OH/ECW, %	16.27±13.63	13.39±15.81	0.169
ECW/TBW	0.50±0.05	0.51±0.05	0.122

* Mann-Whitney *U* test; Median (Interquartile range)

BMI, body mass index; DBP, diastolic blood pressure; ECW, extracellular water; eGFR, estimated glomerular filtration rate; HDL-C, high-density lipoprotein cholesterol; hs-CRP, high-sensitivity C reactive protein; iPTH, intact parathyroid hormone; LAD, left atrial dimension; LAVI, left atrial volume index; LDL-C, low-density lipoprotein cholesterol; LVEDD, left ventricular end-diastolic dimension; LVEF, left ventricular ejection fraction; LVEDV, left ventricular end-diastolic volume; LVH, left ventricular hypertrophy; LVMI, left ventricular mass index; NT-proBNP, N-terminal pro–B-type natriuretic peptide; OH, overhydration; RWT, relative wall thickness; SBP, systolic blood pressure; TBW, total body water.
